# Synthesis of nucleotide–amino acid conjugates designed for photo-CIDNP experiments by a phosphotriester approach

**DOI:** 10.3762/bjoc.9.326

**Published:** 2013-12-18

**Authors:** Tatyana V Abramova, Olga B Morozova, Vladimir N Silnikov, Alexandra V Yurkovskaya

**Affiliations:** 1 Institute of Chemical Biology and Fundamental Medicine, SB RAS, Lavrent’ev Ave, 8, Novosibirsk 630090, Russia; 2 Novosibirsk State University, Pirogova St. 2, Novosibirsk 630090, Russia; 3 International Tomography Center, SB RAS, Institutskaya 3a, Novosibirsk 630090, Russia

**Keywords:** CIDNP, nucleotide–amino acid conjugates, oligonucleotide synthesis, phosphotriester approach

## Abstract

Conjugates of 2’-deoxyguanosine, L-tryptophan and benzophenone designed to study pathways of fast radical reactions by the photo Chemically Induced Dynamic Nuclear Polarization (photo-CIDNP) method were obtained by the phosphotriester block liquid phase synthesis. The phosphotriester approach to the oligonucleotide synthesis was shown to be a versatile and economic strategy for preparing the required amount of high quality samples of nucleotide–amino acid conjugates.

## Introduction

Maintaining the integrity of the genome is of paramount biological importance, since the damage of DNA is considered to cause aging and various degenerative diseases. To prevent the pathological DNA damage, cells evolve the DNA repair machinery, which restores the chemical information encoded in genome. In addition to the enzymatic DNA repair system, a new and quite different repair mechanism, i.e. the non-enzymatic repair, has been discovered. This non-enzymatic system refers to the removal of transient products of the DNA damage like short-lived DNA radicals, with a very high reaction rate by endogenous natural and synthetic compounds, which were extensively studied in chemical systems [1]. This “chemical way” of the DNA repair efficiently competes with the formation of modified sites, which are targets for the enzymatic repair. Since DNA radicals, like all others, are extremely chemically active, they are short-lived, and their concentration is very low to be detected by the conventional electron paramagnetic resonance method. An alternative approach has been developed for the indirect detection of elusive radicals by utilizing nuclear spin hyperpolarization, which occurs in transient radical pairs on a microsecond time scale. Nuclear spin hyperpolarization is preserved in diamagnetic products of chemical reactions for several seconds allowing for NMR detection. This approach is based on the phenomenon of Chemically Induced Dynamic Nuclear Polarization (CIDNP).

Nowadays, the time-resolved variant of CIDNP (TR CIDNP) becomes a powerful method in the investigation of vitally important processes with participation of biological macromolecules [2–5]. The possibility of tracking the electron transfer in the reactions of damaged DNA bases by NMR using in situ photoinitiation and the TR CINDP detection opens a new way to profound investigation of mechanisms of DNA repair. Preliminary experiments in this area [6–7] revealed the necessity to study electron transfer processes in detail using a wide number of specially designed model compound conjugates of the amino acid, nucleotide, and dye residues, where key participants (dye, amino acid, and nucleoside) have to be drawn together mimicking the biologically important DNA repair processes.

To fulfill the requirements of the photo-induced TR CIDNP experiments, a synthetic strategy for obtaining the model conjugates should provide a synthetic versatility, easy scaling up, and high purity of the title compounds. Although there is a number of well-developed methods of the automatic solid phase supported synthesis (SPSS) of oligonucleotide–peptide conjugates [8], this strategy does not ensure the availability of the model conjugates for TR NMR photo-CIDNP experiments due to the lack of versatility and difficulties in scaling up the process. As shown in [9], at least 0.01–0.03 mmol of a compound is required for each photo-CIDNP experiment.

**Figure 1:**
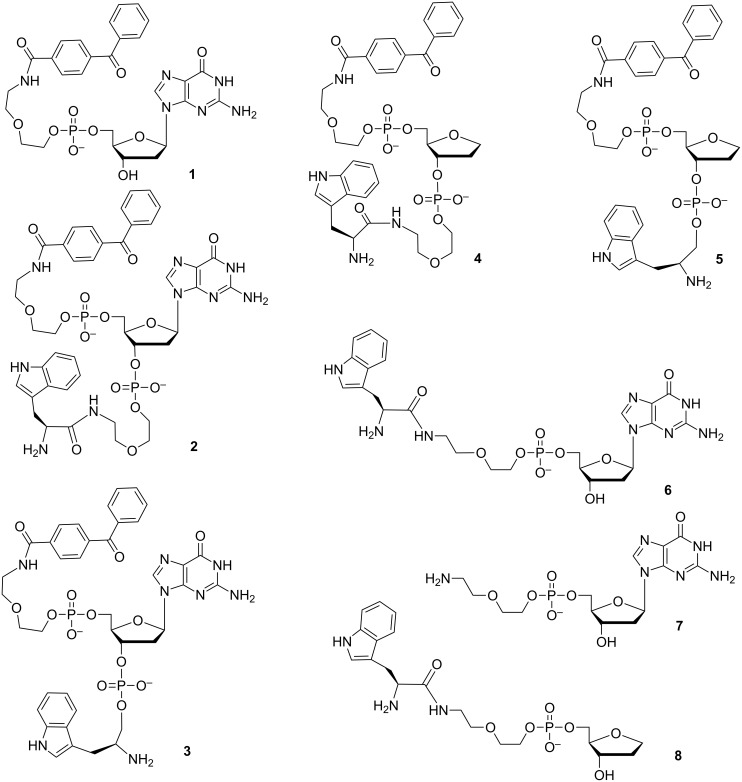
Model compounds for the TR NMR photo-CIDNP experiments: conjugates of 4-benzoylbenzoic acid, 2’-deoxyguanosine, L-tryptophan, and L-tryptophanol.

In this report, we have designed conjugates consisting of amino acid, nucleotide, and dye residues including linkers of different lengths (Figure 1). Target compounds **1**–**8** have been synthesized by the phosphotriester block liquid phase synthesis (LPS).

## Results and Discussion

### Design of model compounds

Our previous studies revealed a great potential of the TR NMR photo-CIDNP technique for the investigation of electron transfer in oxidized peptides and between oxidized DNA nuclear bases and amino acids and small peptides [10–11]. The further development of this work implies the investigation of the electron transfer processes and the detection of elusive radicals in biomolecules using model compounds, where key participants of the reaction (dye, amino acid, and nucleoside) are spatially drawn together mimicking the biologically important DNA repair processes.

Guanosine is the most easily oxidized nucleoside [12] in photoreactions of which pronounced CIDNP effects could be detected [13]. Tryptophan was found as one of the most efficient reducing agents for different protonated forms of oxidized guanosine 5′-monophosphate in a wide pH range [14]. The 2,2’-dipyridyl dye, which is used as a photosensitizer in most of our TR CIDNP experiments, has a disadvantage of a relatively low absorbance at a wavelength of 308 nm at physiological pH. As an alternative, in our investigations water soluble carboxylic derivatives of benzophenone (namely, 4-carboxy-, 3-carboxy- and 3,3’,4,4’-tetracarboxybenzophenone) were used as efficient photoinitiating agents for electron transfer from nucleotides and amino acids [15–18].

Based on the above mentioned reasons, we have chosen 4-benzoylbenzoic acid, 2’-deoxyguanosine, and L-tryptophan [7,9,13–20] as the dye, nucleoside, and amino acid building blocks, respectively, to construct conjugates **1**–**8** (Figure 1) linked in the uniform manner through the phosphate groups.

Conjugates **1** and **6** (Figure 1) are binary molecules combining the dye and nucleoside residues or the amino acid and nucleoside residues, respectively. Conjugates **2** and **3** include all three residues differing in length of the linker between the amino acid and nucleoside residues. Since the amino acid–nucleoside conjugates linked through the phosphamide bond have insufficient stability even at neutral pH [21], we used L-tryptophanol instead of L-tryptophan in conjugates **3** and **5** which do not have the linker group between the amino acid and nucleoside residues to avoid this problem. Phosphodiester linkages in conjugates **1**–**8** are stable in a wide pH range.

Conjugates **4** and **5** containing no guanine nucleobase are control compounds for conjugates **2** and **3**, respectively. Preliminary results revealed a rapid reduction (with the characteristic time of less than 1 μs) of the guanosyl radical by means of electron transfer from the linked tryptophan moiety in conjugate **6**. This efficient reduction was strictly confirmed by comparing CIDNP kinetics of the photoreaction of conjugate **6** (with 2,2’-dipyridyl as photosensitizer) and CIDNP kinetics of the photoreactions of conjugates **7** or **8** under the same conditions.

### Synthesis of model compounds for time resolved photo-CIDNP NMR experiments

It is easy to notice that conjugates **1**–**8** are designed as a kind of di- and trimer nucleotide species. So, the key points of its synthesis are the choice of the strategy (SPSS or LPS) and the method of nucleotide condensation. As mentioned in review [22], LPS is the preferable strategy for obtaining semi-preparative and preparative quantities of short oligonucleotides. This strategy was successfully used in the synthesis of native oligodeoxynucleotides and phosphorothioate hexa-2’-oligodeoxynucleotides by the phosphoramidite method [23–24]. Moreover, LPS was employed for preparing amino acid–nucleotide conjugates [25] and trinucleoside blocks [26] further used in SPSS. The method of nucleotide coupling in LPS presumably depends on the availability of parent compounds and is not limited to the use of more active P(III) nucleotide derivatives.

**Scheme 1:**
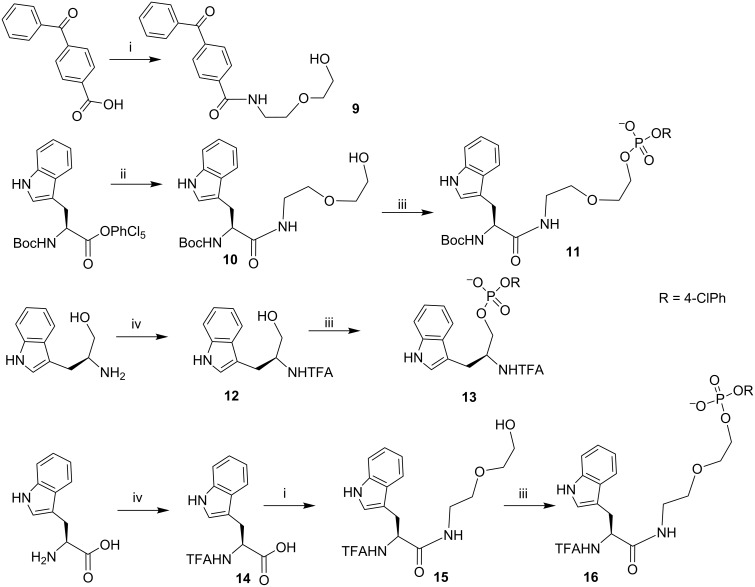
Synthesis of functionalized derivatives of 4-benzoylbenzoic acid (**9**), L-tryptophan (**11**, **16**) and L-tryptophanol (**13**). i) *N,N’*-Dicyclohexylcarbodiimide (DCC), *N*-hydroxysuccinimide (NHS), 1,4-dioxane, then 2(2-aminoethoxy)ethanol); ii) 2(2-aminoethoxy)ethanol), triethylamine (TEA), 1,4-dioxane; iii) *p*-chlorophenyl dichlorophosphate, 1,2,4-triazole, pyridine (Py); iv) ethyl trifluoroacetate, TEA, MeOH.

Taking into account the need for obtaining semi-preparative amounts of conjugates sufficient for TR NMR CIDNP and having in hands the key suitably protected 5’-phosphorylated 2’-deoxyguanosine derivative [27], we used the LPS strategy in combination with the phosphotriester approach for the oligonucleotide synthesis to obtain target conjugates **1**–**8**. The phosphoramidite condensation in LPS usually leads to the lower yields in these reactions due to insufficient stability of active phosphoramidites and the absence of the condensation agent, which dries the solvent and other reagents in the reaction mixture [28]. Functionalized derivatives of commercially available 4-benzoylbenzoic acid, L-tryptophan, and L-tryptophanol, were synthesized by the activation of the carboxylic groups of the suitably protected samples to obtain their active esters followed by the introduction of the additional linker moiety and phosphorylation, if necessary (Scheme 1), according to the well-known procedures [29–31].

**Scheme 2:**
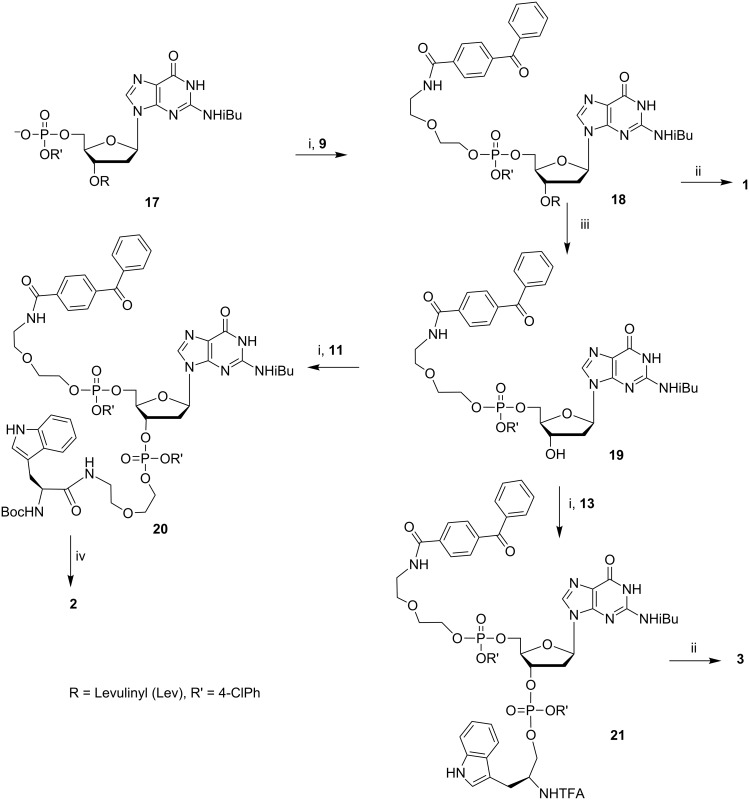
Synthesis of conjugates **1**–**3**: i) 2,4,6-triisopropylbenzenesulfonyl chloride (TPSCl), 1-methylimidazole (MeIm), Py; ii) tetrabutylammonium fluoride (TBAF) in Py/H_2_O, then NH_3_/H_2_O; iii) N_2_H_4_/H_2_O in Py/AcOH; iv) TBAF in Py/H_2_O, then NH_3_/H_2_O, then HC(O)OH.

**Scheme 3:**
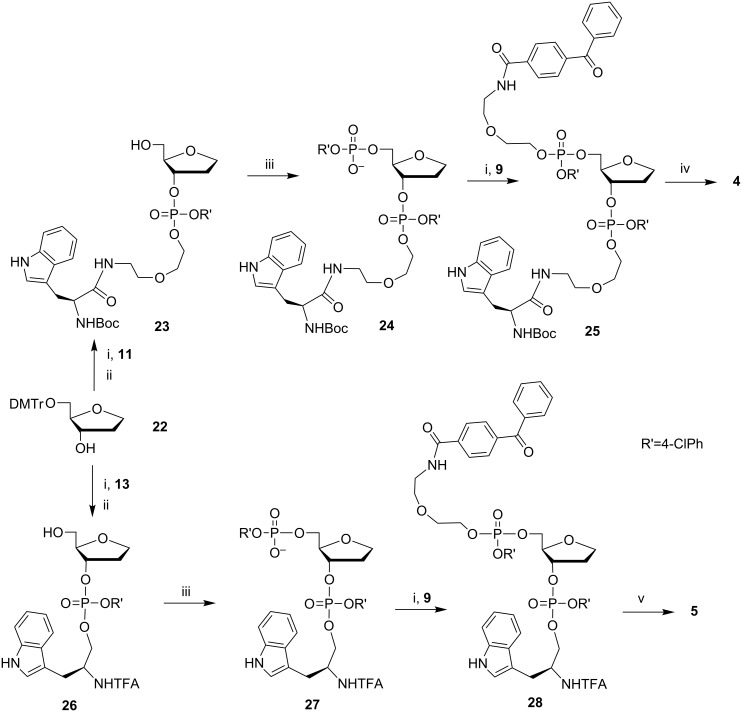
Synthesis of conjugates **4** and **5**: i) TPSCl, MeIm, Py; ii) AcOH/H_2_O; iii) *p*-chlorophenyl dichlorophosphate, 1,2,4-triazole, Py; iv) TBAF in Py/H_2_O, then HC(O)OH; v) TBAF in Py/H_2_O, then NH_3_/H_2_O.

**Scheme 4:**
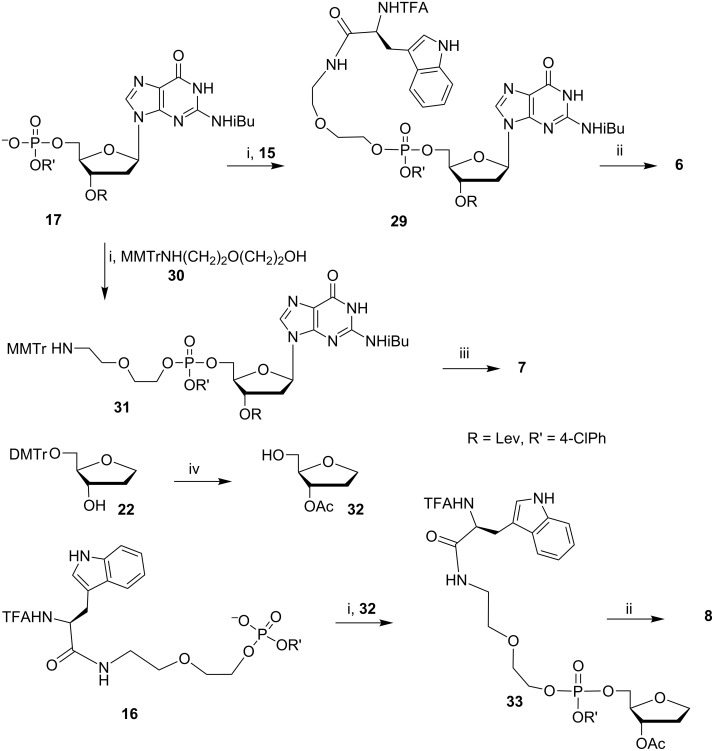
Synthesis of conjugates **6**–**8**. i) TPSCl, MeIm, Py; ii) TBAF in Py/H_2_O, then NH_3_/H_2_O; iii) TBAF in Py/H_2_O, then NH_3_/H_2_O, then AcOH/H_2_O; iv) Ac_2_O, Py, then AcOH/H_2_O.

The synthetic routes for obtaining conjugates **1**–**8** are depicted in Scheme 2, Scheme 3, and Scheme 4. 5-*O*-(4,4'-Dimethoxytrityl)-1,4-anhydro-2-deoxy-D-ribitol (**22**) was obtained as described in [32]. The synthetic approach outlined in Scheme 2, Scheme 3, and Scheme 4 includes the phosphtriester condensation of specially designed and suitably protected blocks containing dye **9**, nucleotide **17** or its abasic analogues **22** and **32,** and the amino acid (or its reduced derivative) **11**, **13**, **15,** and **16**. A series of these blocks and the standard LPS procedures provided a versatile route to obtain semi-preparative amounts (100–200 mg) of the model compounds for the TR CIDNP NMR experiments.

Target conjugates **1**–**8** were obtained after the deprotection of fully blocked derivatives **18**, **20**, **21**, **25**, **28**, **29**, **31,** and **33**. The pretreatment of these phosphotriester derivatives with TBAF under neutral conditions before aqueous ammonia prevented the cleavage of the linker containing the oxoethyl fragment because of β-elimination.

It is worthy to notice that the TBAF treatment is essential when the phosphotriester group contains the aromatic residue (*p*-chlorophenyl in our case), which is much more stable to ammonia than the 2-cyanoethyl group usually used in the phosphoramidite oligodeoxynucleotide synthesis. After the selective cleavage of the aromatic phosphotriester bond by the F^−^ anion, the oxoethyl fragment is quite stable to β-elimination.

We succeeded in the mild deprotection of derivative **20** containing the Boc protective group with formic acid but, to our surprise, we failed in this way when the L-tryptophan moiety was linked to the 5’-OH group of 2’-dG (conjugate **6**). In this case, the deprotection was accompanied by the cleavage of the guanine base at pH 1–5 in aqueous solutions and under the action of a TFA/CH_2_Cl_2_ mixture. So, we replaced Boc by the trifluoroacetyl protective group and synthesized the additional building block **15** to obtain conjugate **6** (Scheme 4).

Keeping in mind the possibility of a further modification of fully protected derivative **31**, we chose the acid labile MMTr protective group for the linker to obtain conjugate **7** because such protection is orthogonal to the protective groups of nucleotide block **17**.

After the deprotection, target conjugates **1**–**8** were purified by the anion exchange and reversed phase chromatography (RPC) at a medium pressure, which provides the high purity of the final products and easy scaling up in laboratory conditions. The homogeneity and structures of all key intermediates and final compounds were confirmed by thin-layer chromatography (TLC), RPC, NMR and mass-spectrometry analysis.

## Conclusion

We designed and synthesized a number of model conjugates to study the role of electron transfer and the elusive radical formation in biologically significant processes. By any standards, the use of the hyperpolarization approach is extraordinary to elucidate the interactions between hidden transient intermediates of nucleosides and amino acids in conjugates, which mimic biologically relevant processes. The liquid phase synthesis (LPS) in combination with the phosphotriester approach appeared to meet the requirements for the in situ NMR detection of the radical reactions with microsecond time resolution by Chemically Induced Dynamic Nuclear Polarization (CIDNP). Based on our fruitful experience in the CIDNP investigation of conjugates **1, 6, 7**, and **8** (will be published soon separately), we are confident that the expansion of this study to other analogous model systems is worth doing for elucidation of complex chemistry, which is developed in nature for maintaining the integrity of the genome encoded in DNA

## Experimental

We used L-tryptophan (Fisher Scientific, USA), Boc-*NH*-L-tryptophan pentachlorophenyl ester (Reanal, Hungary), 2-(2-aminoethoxy)ethanol (Acros Organics, USA). Other reagents were from Sigma-Aldrich, Inc. (USA). Organic solvents were dried and purified by standard procedures. The reaction mixtures were analyzed on a Milichrom A02 analytical chromatograph system (Econova, Russia) using a ProntoSIL 125 C18 column (2 × 75 mm) and gradient of buffer B (0.1 M TEA–AcOH, pH 7.0, 80% acetonitrile) in buffer A (0.1 M TEA–AcOH, pH 7.0, water) with UV detection at 250, 260, 280, and 300 nm. TLC was carried out on Kieselgel 60 F_254_ plates (Merck, Germany) in the proper solvent systems (see below) and spots were visualized by UV irradiation, ninhydrin (for amine groups) or cysteine/aqueous sulfuric acid (for nucleosides and tryptophan) solution. Evaporations were performed under reduced pressure at 40 °C. The preparative silica gel column chromatography was performed using Kieselgel 55–100 μm (Merck, Germany); RPC, a Porasil C 18 (55–105 μm, 125 A) (Waters, USA); and anion exchange chromatography, DEAE Sephadex A-25 (Pharmacia, Sweden). Eluent composition is given in v/v per cent. NMR spectra were acquired on Bruker AM-400 and AV-300 instruments (Bruker, Germany) in appropriate deuterated solvents at 30 °C. Chemical shifts (δ) are reported in ppm relative to the TMS signal. In the case of ^31^P and ^19^F, external standards of 85% H_3_PO_4_ and C_6_F_6,_ respectively, were used. Coupling constants *J* are reported in Hertz. Mass spectra were registered in The Center of Cooperative Use “Proteomics”, Russian Academy of Sciences, on an Autoflex III mass spectrometer (Bruker Daltonics, Inc.) using 2,5-dihydroxybenzoic acid as a matrix (MALDI–TOF) in positive or negative mode.

Compound **22** was synthesized according to the published method [32]. Trifluoroacetamido-*NH*-tryptophan was obtained as described in [33]. The syntheses of compounds **9**, **10**, **12**, **15**, **30,** and **32** were carried out according to the well-known methods [29–30] (see Supporting Information File 1 for details).

### Synthesis of 2-*N*-isobutyryl-3’-*O*-levulinyl-2’-deoxyguanosine 5’-*O*-(*p*-chlorophenyl)phosphate (**17**)

The synthetic scheme and physicochemical data of intermediate compounds see Supporting Information File 1.

2’-Deoxyguanosine 5’-monophosphate (5 g, 12.5 mmol, disodium salt, 0.1 M solution in 20% aqueous EtOH) was converted into NH_4_^+^ form on a DEAE A-25 column (200 mL) by elution with 1 M NH_4_HCO_3_. The desired fractions were evaporated; traces of buffer were removed by coevaporation with water. The last portion of water was added along with TEA (14 mL, 100 mmol). The residue was coevaporated with acetonitrile (3 × 25 mL) and Py (2 × 20 mL) and dissolved in Py (60 mL). Chlorotrimethylsilane (8.2 mL, 6 mmol) was added to the solution under vigorous stirring for 30 min, followed by the addition of isobutyryl chloride (2.7 mL, 26 mmol). After stirring the mixture overnight, it was cooled in an ice bath followed by the addition of water (7 mL) and then (after 10 min) 25% aqueous ammonia. The cooling bath was removed and the reaction mixture was stirred for 1 h at room temperature. After evaporation, the residue was dissolved in water, and the target product was purified by RPC in a linear gradient of EtOH in water (0–20%). The appropriate fractions were pooled and evaporated. After drying, 2-*N*-isobutyryl-2’-deoxyguanosine 5’-monophosphate (5.40 g, 12.0 mmol) was obtained as a cream-coloured powder. Before the next step (protection of the 3’-OH group by the levulinyl residue), the nucleotide with the protected nucleobase was converted to the TEA salt by adding TEA (5 mL, 35 mmol) to its solution in 50% aqueous Py (50 mL), evaporation of resulting mixture and drying the oil residue by coevaporation with acetonitrile (2 × 25 mL) and Py (2 × 20 mL).

Levulinic acid (11.6 g, 100 mmol) and DCC (10.3 g, 50 mmol) was dissolved in diethyl ether (150 mL), and the mixture was stirred for 3 h. After filtration, the diethyl ether was removed from the filtrate by evaporation; the residue was dissolved in Py (50 mL), and the solution was transferred to the flask containing 2-*N*-isobutyryl-2’-deoxyguanosine 5’-monophosphate (TEA salt) and MeIm (4 mL, 50 mmol). The reaction mixture was left for 3 h, followed by adding water (50 mL) and stirring the mixture for 3 h. The reaction mixture was then evaporated several times with water to remove traces of Py. The target product was purified by RPC in a linear gradient of EtOH in water (0–40%). The appropriate fractions were pooled and evaporated. After drying, 2-*N*-isobutyry-3’-*O-*levulinyl-2’-deoxyguanosine 5’-monophosphate (6.0 g, 10.0 mmol) was obtained as a glass-like residue.

2-*N*-Isobutyryl-3’-*O-*levulinyl-2’-deoxyguanosine 5’-monophosphate (6.0 g, 10.0 mmol) was dissolved in CH_2_Cl_2_ (45 mL). Triphenylphosphine (8.0 g, 30 mmol), 2,2′-dithiodipyridine (6.6 g, 30 mmol), and MeIm (4.8 mL, 60 mmol) were added to the nucleotide solution. After 30 min of incubation, the solution of *p*-chlorophenol (6.4 g, 50 mmol) and TEA (21 mL, 150 mmol) in CH_2_Cl_2_ (30 mL) was added, and the reaction mixture was stirred for 3 h. CH_2_Cl_2_ was then evaporated, the residue was dissolved in 50% aqueous Py (100 mL), The solution was washed with diethyl ether (2 × 100 mL), and the product was extracted with a CH_2_Cl_2_/1-butanol mixture (7/3, 2 × 75 mL). The organic layer was evaporated, the residue was dissolved in a minimal volume of 30% aqueous EtOH and loaded on the top of the column containing reversed phase resin equilibrated with water. The target product was purified by RPC in a linear gradient of acetonitrile in water (0–50%). The appropriate fractions were pooled and evaporated. After drying, 2-*N*-isobutyryl-3’-*O-*levulinyl-2’-deoxyguanosine 5’-*O*-(*p*-chlorophenyl)phosphate (1/2 TEA salt, 5.5 g, 7.5 mmol, 60% yield relative to initial 2’-deoxyguanosine 5’-monophosphate) was obtained as a dry glass-like yellowish hydroscopic powder. *R*_f_: 0.63 (iPrOH/H_2_O 4/1); ^31^P NMR (CD_3_OD) 4.75; ^1^H NMR (CD_3_OD) 8.68 (br.s, 1H, N*H*-Gua), 8.14 (s, 1H, *H8*-Gua), 7.16–7.07 (m, 4H, *H*-*p*ClPh), 6.31 (dd, *J =* 5.3, 9.3, *H1’*), 5.48 (m, 1H, *H3’*), 4.35–4.24 (m, 3H, *H-5’,5’’,4’*), 3.22 (q, *J =* 7.3, 3H, *CH*_2_-TEA), 2.89–2.83 (m, 3H, *H2’*, O(O)C*CH_2_*), 2.78 (sep, *J =* 6.8, 1H, *CH*(CH_3_)_2_, 2.64–2.57 (m, 2H, *CH_2_*C(O)CH_3_,), 2.47–2.38 (m, 1H, *H2’’*), 2.20 (s, 3H, C(O)*CH_3_*), 1.32 (t, 6H, *CH_3_*-TEA), 1.21 (d, *J =* 6.8, 3H, CH(*CH_3_*)_2_), 1.19 (d, *J =* 6.8, 3H, CH(*CH_3_*)_2_); MALDI–TOFMS (*m*/*z*): [M + H]^+^ calcd for C_25_H_30_ClN_5_O_10_P, 626.14; found, 626.05; [M + Na]^+^ calcd for C_25_H_29_ClN_5_NaO_10_P, 648.12; found, 648.04.

### General phosphorylation procedure

Compound **10**, **12**, **15**, **23,** or **26** (0.2 mmol) and 1,2,4-triazole (0.08 g, 1.2 mmol) were coevaporated with Py (3 × 1 mL), dissolved in Py (1 mL), and 4-chlorophenyl dichlorophosphate (0.24 mmol, 0.04 mL) was added to the solution under vigorous stirring. After 30 min, the reaction was stopped by the addition of several drops of aqueous 5% NaHCO_3_, and the reaction mixture was evaporated with water to remove Py. The residue was suspended in water, and the target product (compound **11**, **13**, **16**, **24,** or **27**) was purified by RPC in a linear gradient of acetonitrile in water (0–50%). The appropriate fractions were pooled and evaporated. Typical yield was 90% (1/2 Py salt). See Supporting Information File 1 for physicochemical characteristics of compounds **11**, **13**, **16**, **24** and **27**.

### Phosphotriester condensation

The coupling reactions afforded fully blocked derivatives **18**, **20**, **21**, **25**, **28**, **29**, **31,** and **33** were performed according to [34]. After silica gel chromatography, the appropriate fractions were evaporated, and the residue of the fully protected conjugate was subjected to deprotection. Target conjugates **1**–**8** were purified and characterized (see below). The DMTr protective group was removed after the coupling reaction (compounds **23** and **26**) according to [28]. The selective removal of the Lev protective group from compound **18** was performed according to [35]. See Supporting Information File 1 for physicochemical data of compounds **19, 23,** and **26**.

### Deprotection of fully blocked derivatives and purification of conjugates **1**–**8**

Compounds **18**, **20**, **21**, **25**, **28**, **29**, **31,** or **33** after purification by silica gel chromatography (see above) were mixed with 0.33 M solution of TBAF in 50% aqueous Py (pH 7.0, 1.0 mL per 0.05 g of the fully protected derivative) and stirred at 40 °C overnight. The reaction mixtures were then evaporated several times with water to remove Py. The subsequent purification of products by anion exchange chromatography and RPC depended on the combination of the protective groups and a number of negatively and positively charged residues.

#### Conjugate **1**

Partly deprotected by the TBAF treatment conjugate **18** was treated with concentrated (25%) aqueous ammonia for 48 h at room temperature under stirring, and the mixture was evaporated. The residue was dissolved in 40% aqueous EtOH (10 mL per 0.05 g of the fully protected derivative), and the solution was applied to a column with DEAE Sephadex A-25 in 40% aqueous EtOH. Elution was performed with a linear gradient of NH_4_HCO_3_ (0–0.5 M) in 40% aqueous EtOH. The appropriate fractions were pooled and evaporated. Target product **1** was then purified by RPC in a linear gradient of acetonitrile in water (0–20%) in the presence of 0.05 M NH_4_HCO_3_. After drying, 0.120 g of conjugate **1** (0.19 mmol, 37% calcd to **9** taken into the coupling reaction) was obtained. *R*_f_: 0.53 (iPrOH/H_2_O, 4/1); ^31^P NMR (D_2_O) 1.05 (s); ^1^H NMR (D_2_O) 8.01 (s, 1H, *H8*-Gua), 7.91 (dt, *J =* 8.2, 1.6, 2H, *H-*Ar), 7.79 (dt, *J =* 8.2, 1.6, 2H, *H-*Ar), 7.76, (dt, *J =* 7.4, 1.5, 2H, *H-*Ar), 7.71 (tt, *J =* 7.5, 1.2, 1H, *H-*Ar), 7.56 (tt, *J =* 7.4, 1.5, 2H, *H-*Ar), 6.20 (t, *J =* 7.1, 1H, *H1’*), 4.65–4.59 (m, 1H, *H4’*), 4.08–4.02 (m, 1H, *H3’*), 4.05–4.00 (m, 2H, OCH_2_*CH_2_*OP), 3.98–3.92 (m, 2H, O*CH_2_*CH_2_OP), 3.70 (t, *J =* 5.3, 2H, NHCH_2_*CH_2_*O), 3.68–3.64 (m, 2H, *H5’5”*), 3.59 (t, *J =* 5.3, 2H, NH*CH_2_*CH_2_O), 2.54–2.43 (m, 1H, *H2’*), 2.34–2.24 (m, 1H, *H2”*); MALDI–TOFMS (*m*/*z*): [M + H]^+^ calcd for C_28_H_32_N_6_O_10_P, 643.19; found, 643.19; [M + Na]^+^ calcd for C_28_H_31_N_6_NaO_10_P, 665.17; found, 665.21; [M + K]^+^ calcd for C_28_H_31_KN_6_O_10_P, 681.15; found, 681.20; [M − H]^−^ calcd for C_28_H_30_N_6_O_10_P, 641.18; found, 641.62.

#### Conjugate **2**

Partly deprotected by TBAF treatment conjugate **20** was dissolved in a minimal amount of 30% aqueous EtOH and placed on the top of the column containing reversed phase resin equilibrated with water. Elution was performed with a linear gradient of EtOH in water (0–50%) in the presence of 0.05 M NH_4_HCO_3_. The appropriate fractions were pooled and evaporated. The residue was treated with concentrated (25%) aqueous ammonia for 48 h at room temperature under stirring, and the mixture was evaporated. The residue was dissolved in formic acid (1.5 mL). After 3 h, crude conjugate **2** was precipitated by diethyl ether (15 mL), the tube was frozen at −20 °C, and the precipitate was collected by centrifugation. Target product **2** was purified by RPC in a linear gradient of acetonitrile in water (0–30%) in the presence of 0.05 M NH_4_HCO_3_. After drying, 0.06 g of conjugate **2** (0.06 mmol, 15% calcd to **19** taken into the coupling reaction) was obtained. *R*_f_: 0.43 (iPrOH/H_2_O, 4/1); ^31^P NMR (D_2_O) 0.82 (s, 1P), −0.10 (s, 1P); ^1^H NMR (D_2_O) 7.75 (s, 1H, *H8*-Gua), 7.71–7.57 (m, 7H, *H*-Ar), 7.51 (t, *J =* 7.7, 2H, *H*-Ar), 7.40 (d, *J =* 7.8, 1H, *H*-Trp), 7.29 (d, *J =* 7.8, 1H, *H*-Trp), 7.12 (s, 1H, *H*-Trp), 7.04 (t, *J =* 7.8, *H*-Trp), 6.95 (t, *J =* 7.8, *H*-Trp), 5.84 (t, *J =* 7.0, 1H, *H1’*), 4.91–4.83 (m, 1H, *H4’*), 4.29–4.22 (m, 1H, *H3’*), 4.10 (t, *J =* 6.5, 1H, *CH*(NH_2_)CH_2_), 4.00–3.87 (m, 8H, O*CH_2_CH_2_*OP), 3.87–3.44 (m, 10H, NH*CH_2_CH_2_*O, *H5’5”*), 3.42–3.32 (m, 1H, CH(NH_2_)*CH_2_*), 3.24–3.12 (m, 1H, CH(NH_2_)*CH_2_*), *2*.56–2.37 (m, 2H, *H2’H2”*); MALDI–TOFMS (*m*/*z*): [M + H]^+^ calcd for C_43_H_52_N_9_O_15_P_2_, 996.30; found, 996.20; [M + Na]^+^ calcd for C_43_H_51_N_9_NaO_15_P_2_, 1018.29; found, 1018.19.

#### Conjugate **3**

Partly deprotected by TBAF treatment conjugate **21** was subjected to RPC as described for partly deprotected by TBAF conjugate **20**. The residue was then treated with concentrated (25%) aqueous ammonia for 48 h at room temperature under stirring. After evaporation, target product **3** was purified by RPC as described for conjugate **2.** After drying, 0.03 g of conjugate **3** (0.033 mmol, 33% calcd to **19** taken into the coupling reaction) was obtained. *R*_f_: 0.40 (iPrOH/H_2_O, 4/1); ^31^P NMR (D_2_O) 0.76 (s, 1P), −0.66 (s, 1P); ^1^H NMR (D_2_O) 7.76 (s, 1H, *H8*-Gua), 7.66–7.38 (m, 10H, *H*-Ar, *H*-Trp), 7.19 (s, 1H, *H*-Trp), 7.18–7.10 (m, 1H, *H*-Trp), 6.96–6.85 (m, 2H, *H*-Trp), 5.71 (dd, *J =* 6.1, 8.1, 1H, *H1’*), 4.79–4.68 (m, 1H, *H4’*), 4.17–4.10 (m, 1H, *H3’*), 4.08–4.01 (m, 1H, *CH_2_*CH(NH_2_)CH_2_), 4.00–3.86 (m, 4H, O*CH_2_CH_2_*OP), 3.86–3.73 (m, 2H, *CH_2_*CH(NH_2_)CH_2_, CH_2_*CH*(NH_2_)CH_2_), 3.72–3.59 (m, 4H, NH*CH_2_CH_2_*O), 3.58–3.48 (m, 2H, *H5’5”*), 3.24–3.01 (m, 2H, CH_2_CH(NH_2_)*CH_2_*), 2.33–2.18 (m, 1H, *H2’*), 2.06–1.93 (m, 1H, *H2’’*); MALDI–TOFMS (*m*/*z*): [M + H]^+^ calcd for C_39_H_45_N_8_O_13_P_2_, 895.26; found, 895.65; [M + Na]^+^ calcd for C_39_H_44_N_8_NaO_13_P_2_, 917.24; found, 917.51; [M + K]^+^ calcd for C_39_H_44_KN_8_O_13_P_2_, 933.21; found, 933.48; [M − H]^−^ calcd for C_39_H_43_N_8_O_13_P_2_, 893.24; found, 893.14.

#### Conjugate **4**

Partly deprotected by TBAF treatment conjugate **25** was subjected to RPC as described for partly deprotected by TBAF conjugate **20** using gradient of EtOH in water (0–75%). The Boc protective group was removed by formic acid as described for conjugate **2**. The residue was dissolved in 20% aqueous EtOH (10 mL per 0.05 g of fully protected derivative), and the solution was applied to a column with DEAE Sephadex A-25 in 20% aqueous EtOH. Elution was performed with a linear gradient of NH_4_HCO_3_ (0–1.0 M) in 20% aqueous EtOH. After drying, 0.125 g of conjugate **4** (0.15 mmol, 60% calcd to **9** taken into the coupling reaction) was obtained. *R*_f_: 0.59 (iPr/H_2_O, 4/1); ^31^P NMR (D_2_O) 0.75 (s, 1P), −0.09 (s, 1P); ^1^H NMR (D_2_O) 7.54 (d, *J =* 7.8, 2H, *H*-Ar), 7.48–7.15 (m, 9H, *H*–Ar, *H*–Trp), 7.06 (s, 1H, *H*–Trp), 7.00 (t, *J =* 7.2, 1H, *H*–Trp), 6.83 (t, *J =* 7.2, 1H, *H*–Trp), 4.52–4.42 (m, 1H, *H4’*), 4.01 (t, *J =* 7.1, 1H, *CH*(NH_2_)CH_2_), 3.96–3.89 (m, 1H, *H3’*), 3.88–3.80 (m, 2H, *H1’1”*), 3.79–3.50 (m, 8H, O*CH_2_CH_2_*OP), 3.48–3.36 (m, 4H, NH*CH_2_CH_2_*O), 3.33–3.15 (m, 5H, NH*CH_2_CH_2_*O, CH(NH_2_)*CH_2_*), 3.13–2.98 (m, 2H, *H5’5”*), 2.96–2.83 (m, 1H, CH(NH_2_)*CH_2_*), 2.04–1.80 (m, 2H, *H2’2”*); MALDI–TOFMS (*m/z*): [M + H]^+^ calcd for C_38_H_49_N_4_O_14_P_2_, 847.27; found, 847.46; [M + Na]^+^ calcd for C_38_H_48_N_4_NaO_14_P_2_, 869.25; found, 869.43; [M + K]^+^ calcd for C_38_H_48_KN_4_O_14_P_2_, 885.23; found, 885.42; [M − H]^−^ calcd for C_38_H_47_N_4_O_14_P_2_, 845.26; found, 845.60; [M − 2H + Na]^+^ calcd for C_38_H_46_N_4_NaO_14_P_2_, 867.24; found, 867.50.

#### Conjugate **5**

Partly deprotected by TBAF treatment conjugate **28** was subjected to RPC as described for partly deprotected by TBAF conjugate **20** using gradient of EtOH in water (0–65%). The TFA protective group was removed by concentrated (25%) aqueous ammonia for 3 h. After evaporation, target product **5** was purified by anion exchange chromatography as described for conjugate **4**. After drying, 0.09 g of conjugate **5** (0.125 mmol, 50% calcd to **9** taken into the coupling reaction) was obtained. *R*_f_: 0.67 (iPr/H_2_O, 4/1); ^31^P NMR (D_2_O) 0.73 (s, 1P), −0.64 (s, 1P); ^1^H NMR (D_2_O) 7.65 (d, *J =* 8.2, 2H, *H*-Ar), 7.62–7.52 (m, 6H, *H*-Ar), 7.45 (d, *J =* 7.8, 1H, *H*-Trp), 7.43 (t, *J =* 7.9, 2H, *H*-Ar), 7.30 (d, *J =* 7.9, 1H, *H*-Trp), 7.13 (s, 1H, *H*-Trp), 7.05 (t, *J =* 7.5, 1H, *H*-Trp), 6.97 (t, *J =* 7.5, 1H, *H*-Trp), 4.48–4.41 (m, 1H, *H4’*), 3.97–3.83 (m, 4H, *H3’*, *1’1’’*, *CH_2_*CH(NH_2_)CH_2_), 3.83–3.71 (m, 4H, O*CH_2_CH_2_*OP), 3.70–3.56 (m, 6H, *CH_2_*CH(NH_2_)CH_2_, CH_2_*CH*(NH_2_)CH_2_, NH*CH_2_CH_2_*O), 3.55–3.43 (m, 2H, CH_2_CH(NH_2_)*CH_2_*), 3.03–2.95 (m, 2H, *H5’5’’*), 2.01–1.86 (m, 1H, *H2’*), 1.85–1.74 (m, 1H, *H2’’*); MALDI–TOFMS (*m/z*): [M + H]^+^ calcd for C_34_H_41_N_3_O_12_P_2_, 746.22; found, 746.44; [M + Na]^+^ calcd for C_34_H_41_N_3_NaO_12_P_2_, 768.21; found, 768.43; [M − H]^−^ calcd for C_34_H_40_N_3_O_12_P_2_, 744.21; found, 744.41.

#### Conjugate **6**

Partly deprotected by TBAF treatment conjugate **29** was dissolved in 20% aqueous EtOH (10 mL per 0.05 g of the fully protected derivative), and the solution was applied to a column with DEAE Sephadex A-25 in 20% aqueous EtOH. Elution was performed with a linear gradient of NH_4_HCO_3_ (0–0.5 M) in 20% aqueous EtOH. The appropriate fractions were pooled and evaporated. Nucleobase deprotection and the subsequent purification by RPC were performed as described for conjugate **1**. After drying, 0.06 g of conjugate **6** (0.09 mmol, 50% calcd to **15** taken into the coupling reaction) was obtained. *R*_f_: 0.38 (iPr/H_2_O, 4/1); ^31^P NMR (D_2_O) 0.85 (s); ^1^H NMR (D_2_O) 7.78 (s, 1H, *H8*-Gua), 7.36 (d, *J =* 8.1, 1H, *H*-Trp), 7.27 (d, *J =* 8.1, 1H, *H*-Trp), 7.07 (s, 1H, *H*-Trp), 7.04 (t, *J =* 7.5, 1H, *H*-Trp), 6.94 (t, *J =* 7.5, 1H, *H*-Trp), 6.01 (t, *J =* 6.8, 1H, *H1’*), 4.61–4.55 (m, 1H, *H4’*), 4.18–4.13 (m, 1H, *H3’*), 4.10 (t, *J =* 7.0, 1H, *CH*(NH_2_)CH_2_), 4.05–3.94 (m, 2H, CH(NH_2_)*CH_2_*), 3.83–3.69 (m, 2H, *H5’5’’*), 3.44–3.23 (m, 4H, O*CH_2_CH_2_*OP), 3.22–3.04 (m, 4H, NH*CH_2_CH_2_*O), 2.61–2.51 (m, 1H, *H2’*), 2.44–2.35 ((m, 1H, *H2’’*); MALDI–TOFMS (*m*/*z*): [M + H]^+^ calcd for C_25_H_34_N_8_O_9_P, 621.22; found, 621.26; [M + Na]^+^ calcd for C_25_H_35_N_8_NaO_9_P, 643.20; found, 643.24; [M + K]^+^ calcd for C_25_H_34_KN_8_O_9_P, 659.17; found, 659.20.

#### Conjugate **7**

Partly deprotected by TBAF treatment conjugate **31** was dissolved in minimal amount of EtOH and treated with concentrated (25%) aqueous ammonia for 48 h under stirring at room temperature. After evaporation, the residue was dissolved in 40% aqueous EtOH (10 mL per 0.05 g of fully protected derivative), and subjected to anion exchange chromatography as described for conjugate **1**. The appropriate fractions were pooled and evaporated. The residue was treated with 80% aqueous acetic acid (5 mL per 0.1 g of the fully protected derivative) for 30 min, diluted tenfold with water and chilled. The solution was neutralized by addition of concentrated (25%) aqueous ammonia. Target conjugate **7** was purified by RPC as described for conjugate **1**. After drying, 0.015 g of conjugate **7** (0.035 mmol, 25% calcd to **30** taken into the coupling reaction) was obtained. *R*_f_: 0.1 (EtOH); ^31^P NMR (D_2_O) 0.75 (s); ^1^H NMR (D_2_O) 8.04 (s, 1H, *H8*-Gua), 6.29 (t, *J =* 6.7, 1H, *H1’*), 4.73–4.68 (m, 1H, *H4’*), 4.24–4.18 (m, 1H, *H3’*), 4.01 (t, *J =* 4.5, 2H, O*CH_2_CH_2_*OP), 3.84–3.78 (m, 2H, *H5’5’’*), 3.67 (t, *J =* 4.5, 2H, O*CH_2_CH_2_*OP), 3.60–3.46 (m, 2H, NH*CH_2_CH_2_*O), 3.19–3.11 (m, 2H, NH*CH_2_CH_2_*O), 2.88–2.77 (m, 1H, *H2’*), 2.60–2.49 (m, 1H, *H2’’*); MALDI–TOFMS (*m*/*z*): [M + H]^+^ calcd for C_14_H_24_N_6_O_8_P, 435.14; found, 434.99; [M + Na]^+^ calcd for C_14_H_23_N_6_NaO_8_P, 457.12; found, 457.00

#### Conjugate **8**

Partly deprotected by TBAF treatment conjugate **33** was subjected to anion exchange chromatography as described for conjugate **1**. The appropriate fractions were pooled and evaporated. The TFA and Ac protective groups were removed by concentrated (25%) aqueous ammonia treatment for 3 h. After evaporation, target product **8** was purified by RPC as described for conjugate **1**. After drying, 0.026 g (0.055 mmol, 37% calcd to **32** taken into the coupling reaction) was obtained. *R*_f_: 0.32 (EtOH); ^31^P NMR (D_2_O): 0.85 (s); ^1^H NMR (D_2_O) 7.62 (d, *J =* 8.0, 1H, *H*-Trp), 7.52 (d, *J =* 8., 1H, *H*-Trp), 7.31 (s, 1H, *H*-Trp), 7.26 (t, *J =* 7.5, 1H, *H*-Trp), 7.17 (t, *J =* 7.5, 1H, *H*-Trp), 4.30–4.24 (m, 1H, *H4’*), 4.20 (dd, *J =* 8.0, 6.2, 1H, *CH*(NH_2_)CH_2_), 4.02–3.74 (m, 6H, *H1’1’’*, O*CH_2_CH_2_*OP), 3.65–3.05 (m, 9H, *H3’*, *H5’5’’*, NH*CH_2_CH_2_*O, CH(NH_2_)*CH_2_*), 2.17–2.03 (m, 1H, *H2’*), 1.93–1.82 (m, 1H, *H2’*); MALDI–TOFMS (*m*/*z*): [M + H]^+^ calcd for C_20_H_30_N_2_O_9_P, 472.18; found, 472.02; [M + Na]^+^ calcd for C_20_H_29_N_2_NaO_9_P, 494.17; found, 494.02.

## Supporting Information

The synthesis and physicochemical characteristics for compounds **9**, **10**, **12**, **15, 30,** and **32**, the synthetic scheme and physicochemical characteristics for intermediates in the synthesis of compound **17** and the physicochemical characteristics for compounds **11**, **13**, **16**, **19**, **23**, **24**, **26**, and **27** are provided in the Supporting Information.

File 1 Syntheses and characteristics for selected compounds.
