# Supplementary material for: Synthesis of nucleotide–amino acid conjugates designed for photo-CIDNP experiments by a phosphotriester approach
Source: Beilstein J Org Chem. 2013 Dec 18;9:2898–909. doi: 10.3762/bjoc.9.326 (PMC3869286; doi:10.3762/bjoc.9.326)
Supplement: File 1 — Syntheses and characteristics for selected compounds. [file Beilstein_J_Org_Chem-09-2898-s001.pdf]

Supporting Information

for

Synthesis of nucleotide-amino acid conjugates designed for photo-CIDNP experiments by a phosphotriester approach

Tatyana V. Abramova[*1,2], Olga B. Morozova[2,3], Vladimir N. Silnikov[1] and Alexandra V. Yurkovskaya[2,3]

Address: [1]Institute of Chemical Biology and Fundamental Medicine, SB RAS, Lavrent'ev Ave, 8, Novosibirsk 630090, Russia, [2]Novosibirsk State University, Pirogova St. 2, Novosibirsk 630090, Russia and [3]International Tomography Center, SB RAS, Institutskaya 3a, Novosibirsk 630090, Russia

Email: Tatyana V. Abramova[*] - abramova@niboch.nsc.ru

*Corresponding author

Syntheses and characteristics for selected compounds

Synthesis and physicochemical data for compounds 9, 10, 12, 15, 30 and 32

2-[4’-Benzoylbenzamido(2-ethoxy)]ethanol (9)

4-Benzoylbenzoic acid (0.68 g, 3.0 mmol), *N*-hydroxysuccinimide (NHS) (0.40 g, 3.5 mmol) and *N,N’*-dicyclohexylcarbodiimide (DCC) (0.68 g, 3.3 mmol) were dissolved in 1,4-dioxane (10 mL) under stirring. After 2 h, 2(2-aminoethoxy)ethanol (0.37 mL, 3.5 mmol) was added, and stirring was continued for 2 h. After that, reaction mixture was filtrated, precipitate was washed with CH_2Cl_2 (2 × 10 mL), and the filtrate was evaporated. The residue was dissolved in CH_2Cl_2 (50 mL), and the solution was washed with water (2 × 25 mL). Organic layer was dried (Na_2SO_4), filtered, and evaporated. The residue was treated with petroleum ether (30 mL) and dried affording compound **9** as a glass-like residue with quantitative yield (0.94 g, 3.0 mmol). R_f: 0.24 (CH_2Cl_2/EtOH 9.5/0.5); ^{1}H NMR ($CDCl_3$): 7.90 (dt, *J* 8.5, 1.7, 2H, $H_{Benzamido}$), 7.84 (dt, *J* 8.5, 1.7, 2H, $H_{Benzamido}$), 7.80 (dt, *J* 7.1, 2.1, 2H, $H_{benzoyl}$), 7.62 (tt, *J* 7.4, 1.2, 1H, $H_{benzoyl}$), 7.50 (app.tt, *J* 7.7, 1.7, 2H, $H_{benzoyl}$), 6.81 (br.t, *J* 5.5, 1H, *NH*), 3.81-3.77 (m, 2H, CH_2*CH_2*OH), 3.73-3.71 (m, 4H, *CH_2OCH_2*), 3.66-3.63 (m, 2H, NH*CH_2* CH_2); MALDI–TOFMS (*m/z*): $[M + H]^+$ calcd for $C_{18}H_{20}NO_4$, 314.14; found, 314.19; $[M + Na]^+$ calcd for $C_{18}H_{19}NNaO_4$, 336.12; found, 336.18; $[M + K]^+$ calcd for $C_{18}H_{19}KNO_4$, 352.10; found, 352.15.

2-[Boc-*NH*-L-tryptophanamido(2-ethoxy)]ethanol (10)

Boc-*NH*-L-tryptophan pentachlorophenyl ester (1g, 1.8 mmol) was dissolved in 1,4-dioxane (10 mL). 2(2-Aminoethoxy)ethanol (0.25 mL, 2.0 mmol) and TEA (0.28 mL, 2 mmol) were added to the solution. After 1 h, reaction mixture was evaporated. The target product **10** was purified by silica gel chromatography. After drying, 0.66 g (1.68 mmol, yield 93%) was obtained. R_f: 0.50 (CH_2Cl_2/EtOH 9/1); ^{1}H NMR ($CDCl_3$): 8.45

(s, 1H, *NH*-Trp), 7.64 (d, *J* 7.8, 1H, *H*-Trp), 7.33 (dt, *J* 8.0, 0.9, 1H, *H*-Trp), 7.16 (ddd, *J* 8.2, 7.5, 1.1, 1H, *H*-Trp), 7.09 (ddd, *J* 8.2, 7.5, 1.1, 1H, *H*-Trp), 7.03 (d, *J* 2.2, 1H, *H*-Trp), 6.27 (br.t, *J* 6.4, 1H, $NHCH_2CH_2$), 5.26 (br.s, 1H, Boc-*NH*), 4.43-4.31 (m, 1H, *CH*(NH)CH_2), 3.60-3.51 (m, 2H, CH_2CH_2OH), 3.38-3.20 (m, 7H, CH(NH)CH_2, CH_2OCH_2, $NHCH_2CH_2$), 3.17-3.07 (m, 1H, CH(NH)CH_2), 1.40 (s, 9H, *H*-Boc); MALDI–TOFMS (*m/z*): $[M + Na]^+$ calcd for $C_{20}H2_9N_3NaO_5$, 414.20; found, 414.06; $[M + K]^+$ calcd for $C_{20}H_{29}KN_3O_5$, 430.17; found, 430.04.

Trifluoroacetamido-*NH*-L-tryptophanol (12)

L-Tryptophanol (0.64 g, 3.35 mmol) was dissolved in MeOH, TEA (0.7 mL, 5.0 mmol), and ethyl trifluoroacetate (0.6 mL, 5 mmol) were added in the solution. After 3 h, reaction mixture was evaporated. After drying, *N*-trifluoroacetamido-L-tryptophanol 10 (0.94g, 3.35 mmol, quantitative yield) was obtained. R_f: 0.67 (CH_2Cl_2/EtOH 9/1); ^{19}F NMR (CD_3OD): 86.41 (s); ^1H NMR (CD_3OD): 7.63 (dt, *J* 8.1, 1.0, 1H, *H*-Trp), 7.36 (dt, *J* 8.0, 0.9, 1H, *H*-Trp), 7.12 (ddd, *J* 7.8, 7.5, 1.3, 1H, *H*-Trp), 7.10 (s, 1H, *H*-Trp), 7.04 (ddd, *J* 8.4, 7.4, 1.2, *H*-Trp), 4.37-4.24 (m, 1H, CH_2*CH*(NH)CH_2), 3.75-3.59 (m, 2H, CH_2CH(NH)CH_2), 3.16-3.05 (m, 1H, CH_2CH(NH)CH_2), 3.04-2.94 (m, 1H, CH_2CH(NH)CH_2).

2-[Trifluoroacetamido-*NH*-L-tryptophanamido(2-ethoxy)]ethanol (15)

L-Tryptophan was trifluoroacetylated by treatment with ethyl trifluoroacetate according to published procedure [1]. After purification of trifluoroacetylated amino acid by RPC in a linear gradient of acetonitrile (0–30%) in 0.1% aqueous TFA and drying, compound **14** was obtained with a yield 90%. R_f: 0.50 (CH_2Cl_2/EtOH/AcOH 9.5/0.5/0.02); ^{19}F NMR (DMSO-d_6): 85.50 (s); ^1H NMR (DMSO-d_6): 10.88 (s, 1H, COO*H*), 9.78 (d, *J* 8.3, 1H, CH(*NH*)CH_2), 7.55 (d, *J* 8.1, 1H, *H*-Trp), 7.34 (d, *J* 8.1, 1H, *H*-Trp), 7.14 (d, *J* 2.2, 1H, *H*-Trp), 7.07 (ddd, *J* 8.1, 7.1, 1.2, *H*-Trp), 6.99 (ddd, *J* 8.1, 7.1, 1.2, *H*-Trp), 4.55-4.47 (m, 1H, *CH*(NH)CH_2), 3.32 (dd, *J* 14.8 4.2, 1H,

*C*H(NH)*CH₂*), 3.17 (dd, *J* 14.8, 10.3, 1H, *C*H(NH)*CH₂*). Introduction of 2(2-aminoethoxy)ethyl linker was performed as for compound **9**. The target product **15** was purified by silica gel chromatography in a gradient of acetone in CH_2Cl_2 (0–50%). Yield 90%. R_f: 0.23 (CH_2Cl_2/EtOH/AcOH 9.5/0.5/0.02); ^{19}F NMR ($CDCl_3$): 85.91 (s); ^{1}H NMR ($CDCl_3$): 8.41 (br.s, 1H, *NH*-Trp), 7.73 (d, *J* 8.1 1H, *H*-Trp), 7.54 (d, *J* 7.2, 1H, *C*H(*NH*)CH_2), 7.36 (dt, *J* 8.1, 0.9, 1H, *H*-Trp), 7.20 (ddd, *J* 8.1, 7.9, 1.2, *H*-Trp), 7.14 (ddd, *J* 8.1, 7.8, 1.2, *H*-Trp), 7.09 (d, *J* 2.3, 1H, *H*-Trp), 6.06 (t, *J* 5.0, 1H, *NH*CH_2CH_2O), 4.68-4.60 (m, 1H, *CH*(NH)CH_2), 3.61-3.51 (m, 2H, CH_2*CH_2*OH), 3.41-3.22 (m, 6H, *CH_2*O*CH_2*, NH*CH_2*CH_2), 3.22-3.15 (m, 1H, CH(NH)*CH_2*), 3.11 (dd, *J* 14.0, 9.7, CH(NH)*CH_2*).

2-[4’-Methoxytriphenylmethylamino(2-ethoxy)]ethanol (30)

2(2-Aminoethoxy)ethanol (0.10 mL, 1.0 mmol) was dissolved in Py (5 mL). 4-Methoxytriphenylmethyl chloride (0.31 g, 1.0 mmol) was added by portions in 3 h, and the solution was stirred overnight. After that, reaction was stopped by addition of several drops of aqueous 5% $NaHCO_3$, diluted tenfold with CH_2Cl_2 and washed with water (30 mL). The organic layer was dried (Na_2SO_4), filtered, and evaporated. The target product **30** was purified by silica gel chromatography in a gradient of acetone in CH_2Cl_2 (0–25%). After drying, 0.19 g of compound **30** was obtained as a semisolid (0.5 mmol, yield 50%). R_f: 0.43 ((CH_2Cl_2/EtOH 9.5/0.5); ^{1}H NMR (acetone-d_6): 7.48 (dt *J* 8.3, 1.4, 4H, *H*-Ar), 7.40 (dt, *J* 9.0, 2.3, 2H, *H*-Ar), 7.27 (tt, *J* 7.7, 1.7, 4H, *H*-Ar), 7.17 (tt, *J* 7.4, 1.3, 2H, *H*-Ar), 6.84 (dt, *J* 9.0, 2.3), 2H, *H*-Ar), 3.76 (s, 3H, O*CH_3*), 3.62-3.56 (m, 4H, O*$CH_2$$CH_2$*OH), 3.44 (dd, *J* 5.0, 4.3, 2H, NHCH_2*CH_2*O), 2.78 (br.s, 1H, *NH*), 2.30 (t, *J* 5.6, 2H, NH*CH_2*CH_2O).

3-*O*-Acetyl-1,4-anhydro-2-deoxy-D-ribitol (32)

5-*O*-(4’,4’’-Dimethoxytrityl)-1,4-anhydro-2-deoxy-D-ribitol (**22**, 0.12 g, 0.3 mmol) was dissolved in Py (1 mL), and acetic anhydride (0.04 mL, 0.4 mmol) was added. After

16 h, the reaction mixture was evaporated several times with water to remove all Py. The residue was dissolved in AcOH (4 mL) and water (1 mL). After 2 h, the reaction mixture was diluted fivefold with water and evaporated. Evaporation with water was repeated until traces of AcOH would be removed. The residue was dried by coevaporation with acetonitrile (3 x 15 mL) and used without further purification in the coupling reaction with compound **16**.

Synthetic scheme and physicochemical data for intermediates in the synthesis of compound 17

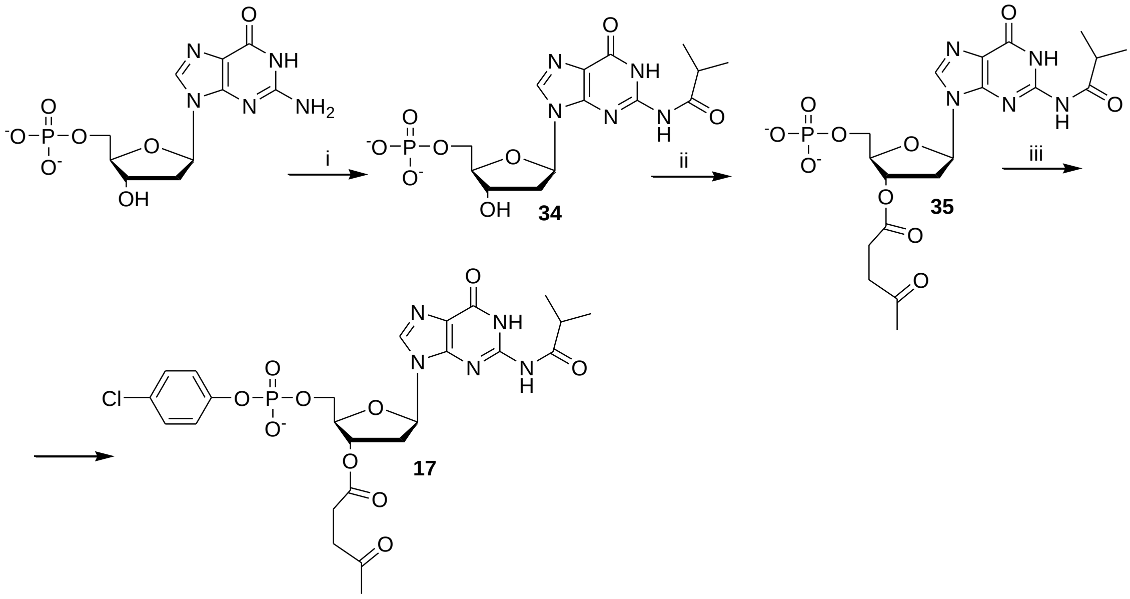

Scheme S1: Synthesis of protected derivative of 2'-deoxyguanosine 5'-phosphate **17**. i) TMSCl, Py, then iBuCl; ii) $(Lev)_2O$, 1-MeIm, Py; iii) Ph_3P, 2,2'$(PyS)_2$, 1-MeIm, then 4-ClPhOH, TEA.

2-*N*-Isobutyryl-2'-deoxyguanosine-5'-phosphate (34)

R_f: 0.62 (iPrOH/H_2O 4/1); ^{31}P NMR (DMSO-d_6): 0.44 (s); ^{1}H NMR (DMSO-d_6): 8.14 (s, 1H, *H8*-Gua), 7.18 (br.s 0.5H, *NH*C(O)CH), 6.64 (br.s 0.5H, *NH*C(O)CH), 6.22 (t, *J* 7.0, 1H, *H1'*), 4.65-4.59 (m, 1H, *H4'*), 4.17-4.06 (m, 1H, *H5'*), 3.99-3.92 (m, 1H, *H3'*),

3.88-3.77 (m, 1H, *H5’’*), 2.82-2.72 (m, 1H, *H2’*), 2.32 (sep, *J* 6.8, 1H, *CH*$(CH_3)_2$), 2.24-2.13 (m, 1H, *H2’’*), 1.12 (d, *J* 6.8, 3H, CH$(CH_3)_2$), 1.11 (d, *J* 6.8, 3H, CH$(CH_3)_2$).

2-*N*-Isobutyry-3’-O-levulinyl-2’-deoxyguanosine-5’-phosphate (35)

R_f: 0.33 (iPrOH/H_2O 4/1); ^{31}P NMR (DMSO-*d*$_6$): 0.18 (s); ^1H NMR (DMSO-*d*$_6$): 8.18 (s, 1H, *H8*-Gua), 7.25 (s 0.5H, *NH*C(O)CH), 7.07 (s 0.5H, *NH*C(O)CH), 6.24 (dd, *J* 9.1, 5.6, 1H, *H1’*), 5.40-5.32 m, 1H, *H3’*), 4.22-4.16 (m, 1H, *H4’*), 4.13-4.04 (m, 1H, *H5’*), 4.01-3.91 (m, 1H, *H5’’*), 3.12-3.00 (m, 2H, OC(O)CH_2), 2.84 (sep, *J* 6.8, *CH*$(CH_3)_2$), 2.77 (t, *J* 6.6, 2H, CH_2C(O)CH_3), 2.72-2.65 (m, 1H, *H2’*), 2.43-2.32 (m, 1H, *H2’’*), 2.14 (s, 3H, C(O)CH_3), 1.12 (d, *J* 6.8, CH$(CH_3)_2$).

Physicochemical data for compounds 11, 13, 16, 19, 23, 24, 26 and 27

2-[Boc-*NH*-L-tryptophanamido(2-ethoxy)]ethyl(*p*-chlorophenyl)phosphate (11)

R_f: 0.10 (iPrOH/H_2O 4/1); ^{31}P NMR (D_2O+CD_3OD): -4.38 (s); ^1H NMR (D_2O+CD_3OD): 8.84 (d, *J* 6.5, 1H, *H*-Py), 8.54 (t, *J* 7.8, 0.5H, *H*-Py), 8.05 (t, *J* 7.0, 1H, *H*-Py), 7.60 (d, *J* 8.2, 1H, *H*-Trp), 7.36 (d, *J* 8.3, 1H, *H*-Trp), 7.31-7.18 (m, 3H, *H*-Trp, *H*-Ar), 7.18-6.97 (m, 4H, *H*-Trp, *H*-Ar), 4.74-4.63 (m, 1H, *CH*(NH)CH_2), 4.39-4.31 (m, 1H, CH_2CH_2OP), 4.08-3.96 (m, 1H, CH_2CH_2OP), 3.85-3.72 (m, 1H, NHCH_2CH_2), 3.54-3.44 (m, 1H, NHCH_2CH_2), 3.29-2.94 (m, 6H, CH(NH)CH_2, CH_2OCH_2), 1.41 (s, 9H, *H*-Boc);); MALDI–TOFMS (*m*/*z*): $[M - H]^-$ calcd for $C_{26}H_{32}ClN_3O_8P$, 580.16; found, 579.60.

Trifluoroacetamido-*NH*-L-tryptophanolyl(*p*-chlorophenyl)phosphate (13)

R_f: 0.27 (CH_2Cl_2/EtOH 8/2); ^{19}F NMR (CD_3CN): 88.16 (s); ^{31}P NMR (CD_3CN): -4.70 (s); ^1H NMR (CD_3CN): 9.18 (s, 1H, *NH*-Trp), 8.88 (d, *J* 7.9, 1H, CH_2CH(*NH*)CH_2), 8.59 (d, *J* 4.5, 1H, *H*-Py), 8.40 (t, *J* 7.8, 0.5H, *H*-Py), 7.86 (dd, *J* 7.8, 4.5, 1H, *H*-Py),

7.62 (d, *J* 8.0, 1H, *H*-Trp), 7.40 (dt, *J* 8.2, 1.8, 1H, *H*-Trp), 7.26 (dt, *J* 8.9, 5.0, 2H, *H*-Ar), 7.20-7.01 (m, 5H, *H*-Trp, *H*-Ar), 4.38-4.26 (m, 1H, CH_2*CH*(NH)CH_2), 4.18-3.98 (m, 2H, *CH_2*CH(NH)CH_2), 3.08-2.94 (m, 2H, CH_2CH(NH)*CH_2*).

2-[Trifluoroacetamido-*NH*-L-tryptophanamido(2-ethoxy)]ethyl(*p*-chlorophenyl)phosphate (16)

R$_f$: 0.10 (CH_2Cl_2/EtOH 8/2); ^{19}F NMR (acetone-*d*$_6$): 88.60 (s); ^{31}P NMR (acetone-*d*$_6$): -5.41 (s); ^{1}H NMR (acetone-*d*$_6$): 8.73 (s, 1H, *NH*-Trp), 8.72 (d, *J* 6.0, 1H, *H*-Py), 8.27 (t, *J* 7.6, 0.5H, *H*-Py), 7.87 (br.t, *J* 6.5, 1H, CH(*NH*)CH_2), 7.77 (dd, *J* 7.6, 6.0, 1H, *H*-Py), 7.63 (d, *J* 8.0, 1H, *H*-Trp), 7.32 (d, *J* 8.0, 1H, *H*-Trp), 7.31-7.25 (m, 4H, *H*-Ar), 7.22 (s, 1H, *H*-Trp), 7.04 (ddd, *J* 8.1, 7.1, 1.3, 1H, *H*-Trp), 6.97 (td, 7.8, 0.9), 4.78-4.69 (m, 1H, *CH*(NH)CH_2), 4.18-4.08 (m, 2H, CH_2*CH_2*OP), 3.62-3.53 (m, 2H, *CH_2*O*CH_2*), 3.51-3.19 (m, 6H, *CH_2*O*CH_2*, NH*CH_2*CH_2), CH(NH)*CH_2*).

2-*N*-Isobutyryl-2'-deoxyguanosine 5'-*O*-{2-[4''-benzoylbenzamido(2-ethoxy)]ethyl}(*p*-chlorophenyl)phosphate (19)

R$_f$: 0.26 (CH_2Cl_2/EtOH 9/1); MALDI-TOFMS (*m*/*z*): $[M + H]^+$ calcd for $C_{38}H_{41}ClN_6O_{11}P$, 823.23; found, 823.15; $[M + Na]^+$ calcd for $C_{38}H_{40}ClN_6NaO_{11}P$, 845.21; found, 845.14; $[M + K]^+$ calcd for $C_{38}H_{40}ClKN_6O_{11}P$, 861.18; found, 861.11; $[M\text{-}H]^-$ calcd for $C_{38}H_{39}ClN_6O_{11}P$, 821.21; found, 821.31.

1',4'-Anhydro-2'-deoxy-D-ribityl-3'-*O*-{2-[Boc-*NH*-L-tryptophanamido(2-ethoxy)]ethyl(*p*-chlorophenyl)}phosphate (23)

R$_f$: 0.50 (CH_2Cl_2/EtOH 9/1); ^{31}P NMR ($CDCl_3$): -6.66, 7.08 (2s, two diastereomers).

5'-*O*-[(*p*-Chlorophenyl)phospho]-4'-anhydro-2'-deoxy-D-ribityl-3'-*O*-{2-[Boc-*NH*-L-tryptophanamido(2-ethoxy)]ethyl(*p*-chlorophenyl)}phosphate (24)

R$_f$: 0.07 (CH_2Cl_2/EtOH 9/1); ^{31}P NMR ($CDCl_3$): -6.53 (br.s, 1P), -6.92, -7.36 (2s, 1P, two diastereomers).

1,4-Anhydro-2-deoxy-D-ribityl-3-*O*-[Trifluoroacetamido-*NH*-L-tryptophanolyl(*p*-chlorophenyl)]phosphate (26)

R_f: 0.12 (CH_2Cl_2/EtOH 9.5/0.5);^{19}F NMR ($CDCl_3$): 86.01, 85.99 (2s, two diastereomers); ^{31}P NMR ($CDCl_3$): -6.03, -6.53 (2s, two diastereomers).

5'-*O*-[(*p*-Chlorophenyl)phospho]-4'-anhydro-2'-deoxy-D-ribityl-3'-*O*-[Trifluoroacetamido-*NH*-L-tryptophanolyl(*p*-chlorophenyl)]phosphate (27)

R_f: 0.14 (CH_2Cl_2/EtOHl 9/1); ^{19}F NMR (CD_3CN): 88.65, 88.64 (2s, two diastereomers); ^{31}P NMR (CD_3CN): -5.80 (br.s, 1P), -6.56, -6.76 (2s, 1P, two diastereomers).

References

1. Curphey, T. J. *J. Org. Chem.* **1979,** *44,* 2805-2807.
